# Opinion of Polish Patients with Epilepsy on Generic Medications

**DOI:** 10.3390/healthcare11202717

**Published:** 2023-10-12

**Authors:** Katarzyna Swakowska, Urszula Religioni, Anna Staniszewska

**Affiliations:** 1 Civitas 24 GmbH, 55122 Mainz, Germany; swakowskakasia@gmail.com; 2 School of Public Health, Centre of Postgraduate Medical Education of Warsaw, 01-826 Warsaw, Poland; urszula.religioni@gmail.com; 3 Department of Experimental and Clinical Pharmacology, Medical University of Warsaw, 02-097 Warsaw, Poland

**Keywords:** generic medicines, opinions, patients, epilepsy, Poland

## Abstract

Aim: The majority of medicines used in Poland are generic drugs and substitutions of the originals. The objective of this study was to obtain information on the current knowledge about generic medicines among Polish patients with epilepsy. Material and Methods: The conducted study was based on a self-developed questionnaire. The questionnaire consisted of 26 questions, including the questions regarding the respondents’ knowledge about generics, their previous experience, and the factors behind these choices. Results: Overall, 1220 questionnaires were analyzed. Among all patients, 66.4% reportedly had heard about generics. Of these, 61.5% of patients had used generic drugs in the past. A significant proportion of participants had never been recommended to switch to a generic medicine by their healthcare professional (23% physicians and 13.9% pharmacists). Statistically, relevant differences were observed regarding the respondents’ kind and level of education, place of residence, and net income per household. Conclusions: Significant gaps were identified in the knowledge and perceptions among patients regarding generic medicines, especially in relation to their efficacy and safety. Efforts must be directed towards increasing public awareness of generic medicines and there should also be a focus on educating patients about generic medicines.

## 1. Introduction

Generic medicines are extremely popular in Poland and the share of generic medicines in Poland is the highest in the European Union [1]. It is proved that the costs of medicine are one of the main expenditures of healthcare systems [2] and of households in different countries [3,4,5]. A generic substitution allows patients to buy much cheaper medicines. Different studies show that generic substitution can significantly reduce the cost of medicines to the healthcare systems [6,7]. The average price of a generic in Poland is 2.6 times less than the price of a brand-name drug [8]. There have been many recommendations about the use of generic medicines and increasing the equivalence of these medicines has been linked to the desire to minimise healthcare expenditure.

The more generic medicines on the market, the more price competition in the pharmaceutical markets leads to significant price reductions (for generics on average by 50%) [9]. This was covered in the report from the Commission to the Council and the European Parliament on the enforcement of competition rules in the pharmaceutical sector (2009–2017), 2019 [9].

However, we should bear in mind that any substitution of a generic drug may be associated with a change in the efficacy and safety of pharmacotherapy [10]. Substituting original medicines with the generic ones is only possible once bioequivalence of the medicines has been demonstrated [11]. Medicinal products are considered to be bioequivalent when they contain the same active substance and their bioavailability at the same molar dose is similar to the extent that their efficacy and safety are essentially similar. Therefore, bioavailability is important, which indicates the rate and extent of absorption of a medicinal product from the formulation into the general circulation. It is characterized by three pharmacokinetic parameters: maximum drug concentration (Cmax), time to reach Cmax (tmax), and area under curve (AUC) [12]. However, the pharmacokinetic characteristics of the target population may be different from those of the bioequivalence study. In this case, the attempt to switch may be questionable, particularly in groups of patients with variable pharmacokinetics and in the presence of conditions that may adversely affect the pharmacokinetics of the medicinal products. Patients in whom special caution should be exercised due to variable pharmacokinetics include infants, children, pregnant women, and the elderly [10]. Unawareness and lack of acceptance of generic drugs are the main barriers to their use. The aim of this study was to assess knowledge on generic medications of patients with epilepsy. The specific objective was to examine which socio-demographic variables are independently related to the acceptance of generic drugs.

## 2. Materials and Methods

Taking into account the extensive literature on generic medicines, a questionnaire was developed to assess the knowledge and perception of generic medicines. The questionnaire consisted of three parts:

1. socio-economic data;
2. generic knowledge;
3. experiences with generic medicines.

The questions in the survey focused on generic medicines in general, rather than on the generic antiepileptic drugs. The questionnaire consisted of 26 questions, including questions regarding the respondents’ knowledge about generics (8 questions listed in Table 1, their previous experience (8 questions listed in Table 2), and the factors behind these choices (socio-demographic and clinical parameters—9 variables included in Table 3 and age). The study used the CAWI (computer assisted web interview) technique. The study was anonymous and participation was voluntary. Taking part in the study was free of charge. The completion of the survey was deemed to be an agreement of consent from the participants. The inclusion criterion was as follows: the age of 18 or over. The study was conducted between January 2022 and June 2023 on selected online websites, a collective forum for people suffering from epilepsy. The time to complete the survey was unlimited, but this survey took, on average, 10 to 13 min to complete. A total of 1220 out of 1500 patients participated in the study, giving a response rate of 78.7%.

### 2.1. Statistical Analysis

The data were analyzed through the statistical package StatSoft. Inc STATISTICA software (version 13.03) and Excel spreadsheet. Quantitative variables were characterized by mean, standard deviation, median, mode, and minimum and maximum value (range). Variables of the qualitative type were presented by means of the number and percentage values. Chi square tests of independence were used for categorical variables. Additionally, a correlation analysis was used to calculate the Pearson and/or Spearman correlation coefficients. Statistically significant values were *p*-values < 0.05. The respondents’ answers were correlated with socioeconomic characteristics of the respondents: age, sex, marital status, level and kind of education, place of residence, and net income-per-household-member.

### 2.2. Ethical Approval

The study was approved by the Ethics Committee of the Medical University of Warsaw (ref. no. AKBE/159/16, date 11 October 2016).

## 3. Results

### 3.1. Characteristics of Participants

The survey was completed by 1220 participants, 900 (73.8%) were females. Mean age of respondents was 38.2 yrs (SD = 17.3; median 32; mode 23; min. 20, max. 88). Most of the respondents (n = 510; 41.8%) lived in a marriage relationship. The majority (n = 940; 77.1%) of the individuals who took part in the survey lived in a city >500 thousand residents. Education-wise, almost ¾ (n = 900; 73.8%) of the total respondents were university educated, while 210 (17.2%) included a medical background. The Polish Zlotych (PLN) 2001–3000 was the most declared average monthly income per respondent (n = 340; 27.9%). An equal number of respondents (n = 430; 35.3% each), were chronically ill with a disease other than epilepsy and took medicines other than antiepileptic drugs in relation to a chronic disease. More than half (n = 590; 56.6%) of the respondents affirmed that they benefited from both public and private health units, and 30 (2.5%) stated that they were not treated at any type of healthcare facility. Table 1 presented characteristics of the respondents.

### 3.2. Knowledge about Generic Medicines

In this study, only 810 (66.4%) patients had already heard about generic medicine, and 410 (33.6%) had never heard about these medications. Among the respondents, only 570 (46.7%) knew the term generic medicine. Most respondents (n = 420; 34.4%) obtained information on generic medicines from medical practitioners. More than half of the surveyed population (n = 620; 50.8%) agreed with the statement that a generic medicine could be produced free of charge after the expiry of the patent protection and must be similar to the original drug in order to obtain the same therapeutic effect. The overwhelming percentage of respondents (n = 790; 64.8%) believed that the generic drug contains the same substance as the branded medicine.

An equal number of respondents (n = 640; 52.5%) each stated that generic medicines were of just as good quality and were just as safe as reference medicines. Almost ¾ of the respondents (n = 890; 730%) considered generic medicines to be less expensive than reference medicines. Table 2 presented knowledge about generic medicines of the respondents.

### 3.3. Experience with Generic Medications

Less than half of respondents (n = 470; 38.5%) claimed they had never used a generic medicine or did not know about them.

Among respondents who had an experience with generic medicines, only 2.5% (n = 30) of the study population always buy generic drugs.

When asked whether generic medicines are prescribed by doctors, nearly one third of patients (n = 400; 32.8%) were not sure.

The pharmacists always recommended generics for only 1.6% (n = 20) of the respondents.

Most of the patients never asked his/her doctor or pharmacist (n = 600; 49.2%) to prescribe/distribute generic medicines.

More than half (n = 660; 54.0%) of the respondents stated that they buy generic drugs because of the price.

Almost none of the respondents who took generic medicines (n = 730; 59.9% of all participants) did not feel that switching from a brand name to a generic medicine changed the outcome of therapy.

It found that patients who took generic medicines did not see increased rates of medicine-related side effects (n = 690; 56.6% of all participants).

The practice-related questions and the responses of the participants are summarized in Table 3.

### 3.4. Results of Statistical Analysis

The aim of the study was also to determine the relationship between the answers to the questionnaire and the sociodemographic characteristics of the patients (Table 4).

There was a statistically significant association in terms of the knowledge of generic medicines and the kind of education of the respondents. The respondents with medical education have better knowledge on generic medicines (in all questions *p* < 0.02)—Table 5, Table 6, Table 7 and Table 8.

The level of education statistically differentiated the choice and knowledge of respondents. Individuals with a university degree significantly often:

− claimed that the generic medicine is good quality compared to the branded medicine (*p* = 0.000);
− claimed that the generic medicine is as safe as the branded medicine (*p* = 0.000);
− claimed that the price of the generic medicine is less than the branded medicine (*p* = 0.000);
− have taken generic medicines (*p* = 0.044);
− were buying generic medicines (*p* = 0.001).

Statistically significant associations were found between the place of residence of the respondents and choice, and the answers to the question about the generic medicines. The respondents who lived in a city >500 thousand residents significantly often:

− claimed that the generic medicine is of good quality compared to the branded medicine (*p* = 0.013);
− claimed that the price of the generic medicine is less than the branded medicine (*p* = 0.002);
− have taken generic medicines (*p* = 0.009).

Net income per household was another factor which significantly differentiated respondent choices. Individuals with up to PLN 2000 net income per household significantly more often declared that they had heard about generic medicine (*p* = 0.049)—Table 9.

No statistically significant differences were found between other variables (*p* > 0.05).

## 4. Discussion

The Polish study on patients’ opinions about generic medicines was conducted in 2011 among 500 patients [13], and in 2013 among a population of 1000 patients [14], while the discussed study was conducted in January 2022–June 2023, and 1220 patients took part in it.

Among the respondents in our own study, most of the respondents had already heard about generic medicines. Worse than this, it was noted in Auckland, New Zealand, that only 51% of their respondents had heard of the term “generic medicines” [15], and in Malaysia, it was noted that 85.8% did not know the term “generic medicines” [16]. However, this aspect was noted to be better in studies conducted in Brazil. In the study by Lira et al., almost all respondents had already heard about generic medicine [17], and similarly in the study by da Rocha et al. [18].

Almost half of the respondents to the current survey declared that they had some knowledge of reconstructive drugs. Findings similar to those presented in this study were described in a study conducted in Malaysia of 216 people, 32.5% of whom stated that they knew what generic drugs were [19].

A generic drug is defined as a drug, the production of which is possible at the time of expiry of the patent protection of the original drug [20]. In this study, only half of the respondents knew about that. More than half of the respondents said that generic medicines were comprised of the same substances as the reference medicines. Likewise, in the study by Lira et al. more than half of the respondents defined generics as medications with the same active ingredient(s) as the original medicines [17]. A generic medicine is a product that is launched with no intellectual property or other protection after the protection expires on the originator medicine. The original and generic medicine may differ in name, manufacturer, and price. However, the active substance contained in them, which is responsible for the action of the drug, and its amount, will always be the same [21].

Generic medicines were considered “same in quality” to brand-name products by 52.5% of the respondents. In 1994, in the study by Muirhead, generic products were considered “equal in quality” to brand-name products by 29% of consumers, and 45% indicated that the two were “about the same” [22]. However, 14.4% of the respondents in the study by Lira et al. thought that generic drugs were inferior to the original drugs [17]. Fortunately, in this study, the knowledge of the respondents was better than that noted in other countries, and only 10.7% of the respondents thought that generic drugs were of poorer quality than the reference drugs. Similarly, the difference in quality between generic and original drugs is not seen by 11.6% of the study group analyzed by Grzywinska [13]. Among the respondents, 640 (52.5%) thought that the replacement drugs were as safe as the reference drugs, however, in the study by Lira et al., it was 75.2% [17]. Generic medicines have the same level of effectiveness and quality as brand-name drugs [21].

Generic medications, typically, cost less. When asked about price, almost 3/4 of the respondents stated that generic medications cost less than the branded medicines. In the study by Naing et al., the vast majority of the respondents were unaware of price differences between generic and reference medicines [16], however, in the study by Lira et al., 88.8% stated that generic drugs are less expensive than the reference drugs [17]. While asking the respondents about the reasons for generic substitution, as many as 660 per 1220 respondents (which is 54.1%) mentioned that they thought it was because the generics were cheaper. Generic alternatives are often cheaper than brand-name medicines because the manufacturers have not spent money on research and development of the medicine or buying the rights to sell it [23]. In Poland, most patients are directed by price in choice of medicines [24,25].

The question “Have you already taken generic medicines?” was aimed at verification of the number of patients who reach for these medicines. This question also qualified the respondents for answering the subsequent questions. Analysis of responses indicated that 61% patients admitted that they used generic medicines but 38.52% of respondents claimed that they had never used or do not know about the cheaper generic substitutes for original drugs. This result is similar to that obtained in the other study conducted in Poland in 2015 [14]. Among patients who took generic medications, as many as 360 respondents, i.e., 29.5%, sometimes bought generic drugs. In Poland, the use of generics is high. According to 2017 data, among the reimbursed prescription medicines, the share of generics was 27% by value and 89% by volume in hospitals and, respectively, 66% and 76% in outpatient pharmacies [26].

In Poland, physicians are able to make individual choices for prescribing drugs, and these choices are largely based on brand. In Poland, it is the doctor who decides which medicine he will prescribe to a patient. In our own study, almost ¼ of patients declared that their doctor never offered them a generic drug during the visit. On the other hand, in another study conducted in Poland, of a group of 119 doctors, the results showed that almost 70% of the surveyed doctors (n = 83) admitted that they prescribed mostly generic drugs to their patients [13]. In Greece, overall, 75% of physicians claimed that they were not influenced by the sales representatives from drug companies and that Greek patients do not interfere with their prescribing, but they often complain about the drug costs [27].

In Poland, there is no obligation to replace original drugs with generics; nevertheless, the pharmacists have the right to switch from branded to generic medicines unless the prescriber has specified otherwise [28,29]. In the present study, the pharmacists sometimes recommended generics for 42.6% of the respondents. Whereas, Grzywinska evaluated community pharmacists’ practices on generic medicines in Poland [13], and of the pharmacists surveyed, 65% recommended generics over original brands. It was found that 66% of pharmacists recommended the substitution of a prescribed branded drug by a different form of the same active substance often, or very often, but only 25.41 per cent of respondents replied that their doctors talked to them about generic medications and sometimes prescribed these medicines. A study conducted in Poland, “Factors affecting the opinions of family physicians regarding generic drugs—a questionnaire-based study”, by Lewek et al., showed that 73.0% of physicians were considering prescribing generics and 71.1% regularly informed patients of this possibility. The physicians who considered generics when prescribing a drug tended to report doing so either often or always (in 50–100% of cases; *p* < 0.001) and were more ready to inform patients about generic substitution (*p* < 0.001) [30]. It should be noted that generic substitution of branded products has played an important role in limiting the cost of medicines.

The patients themselves may also request generic versions of prescribed medications when they are either with the doctor, or when they visit the pharmacy to collect their prescription, or when they buy over-the-counter drugs. In our own study, only 4.1% (n = 50) of patients always request a switch from an original drug to the generic drug from their doctor or pharmacist.

The results in the previously mentioned study are different; about ¼ of patients asked their doctor for a cheaper drug often or very often, while 15.2% of patients never asked a doctor for a generic drug [13]. In the same study, doctors and pharmacists were also asked how often patients themselves ask for a generic substitution. It turned out that, according to physicians, patients ask them to replace the drug with a cheaper equivalent often or very often in almost half of the cases, i.e., in 47.9% of cases, while ¼ of the surveyed pharmacists (25%) stated that patients themselves rarely ask them to change an innovative drug to generic [13]. Another Polish survey conducted among 22 pharmacists showed that 59% of pharmacy employees stated that patients “never” on their own initiative sought information about the possibility of replacing the drug with its cheaper equivalent [31].

Most of the respondents in this study (n = 420; 34.4%) obtained information on generic medicines from medical practitioners. In the study conducted by Kjoenniksen et al., 24% of participants indicated that their physicians had given them information about generic substitution, while a larger proportion (53%) indicated that the pharmacist had done so [32]. Grzywinska has shown that 30% of pharmacists declared that patients ask them often or very often about the difference between generics and branded medicines [13]. Firstly, the healthcare professionals are a reliable source of information of generic substitution. Secondly, studies conducted in Poland show that the pharmacists have a position of public trust and are fully competent to provide information on medications in patients’ opinions [24]. Similarly, most of the patients trust their doctors [33].

Among patients with previous experience with generic switching, only 1.6% of respondents felt the impact of changing brands of medicines and only 4.9% felt side effects. From the manuscript “A review of patient perspectives on generics substitution: what are the challenges for optimal drug use”, it follows that between 8–34% of patients reported poorer effects and/or new side effects after a change—except for antiepileptic drug users of which the number of reports was even higher [34].

Out of various factors that may affect knowledge of generic drugs and their use, high education, medical education, place of residence (city > 500 thousand residents), and net income per household (up to PLN 2000 = about 500 USD) were statistically significant. The results were consistent with a study conducted by Iosifescu et al., where it was observed that negative beliefs about generic medications were associated with lower education and low income [35]. Similarly, a meta-analysis by Dunne and Dunne evaluating perceptions of physicians, pharmacists, and patients found that patients with less education were more skeptical of generic medications [36].

In this study, there was no statistically significant relationship between the sex of respondents and responses of participants about generic medicines. Other studies were in line with our findings [13,34,35,37].

Additionally, in our own study, there were no statistically significant differences between responses of respondents in terms of age, marital status, chronic illness other than epilepsy, long-term use of medication other than antiepileptic drugs, and healthcare facility where respondents receive outpatient care.

Generic substitution is a common practice in most healthcare systems [38]. It is vital to remember that generic substitution has its pros and cons. On the one hand, the usage of generic medications is economically justified, but, on the other hand, there appear to be concerns about the safety and effectiveness of treatment. However, many opinions on this issue are ambiguous or even contradictory. According to Krauss and Privitera’s study, generic substitution appears to be safe for antiepileptic drugs [39]. However, Wilner’s study [40] showed that after switching from the original drug to a generic, as many as 81.4% of neurologists reported an increase in seizures (67.8%) or toxic symptoms (56%) in their epilepsy patients. In addition, when switching from one generic drug to another, the proportion was 32.5% and 26.6%, respectively. In this case, not only the health of patients deteriorated, but also the cost of their therapy increased. Thus, generic substitution is not always effective and cost-effective. For levothyroxine, a thyroid medicine, the use of its substitutes was associated with a risk of side effects [41]. In conclusion, thinking about switching from an original drug to a generic drug should take into account, among other factors: the patient’s age; pharmacogenetic factors; co-existing diseases; underlying disease; bioequivalence studies; or the use of other drugs [10]. In Poland, studies have been conducted on opinions on generic drugs among medical staff, however, not many studies have been conducted among patients in Poland.

This study has some limitations. The study involved a large group of people with higher education, including medical education, which may not reflect the opinion of the general public about generic drugs. It is very similar when it comes to a place of living. The questions in the survey concerned generic medicines in general, not generic anti-epileptic drugs. However, it can provide useful data for doctors, pharmacists, and health policy makers to further improve the use of generic medicines by considering the patients’ perspectives. However, the fact that this is the first, and, so far, the only, study on epilepsy patients’ knowledge on generic medications conducted in Poland is worth a mention. Hence, it should constitute a foundation for further detailed research. This study has limitations due to its being confined to a single group—patients with epilepsy—and lack of a control group. Further research should be performed to compare the results of substitution of generics for brand-name drugs in the treatment of patients with epilepsy.

## 5. Conclusions

The presented study allowed the drawing of the following conclusions. Epilepsy patients’ knowledge concerning generic medicines was moderate. According to the study findings, there were gaps in the epilepsy patients’ knowledge of generic medicines. Incorrect information was common among epilepsy patients about the safety, efficacy, and quality of generic medicines. Despite the knowledge on generic medicines, our respondents showed mixed beliefs about generic medicines. Lack of knowledge about generic medicines may influence patients’ negative perceptions of generic medicines. Thus, an awareness campaign would be needed to expand patients’ knowledge about generic medicines.

## Figures and Tables

**Table 1:** Knowledge about generic medicines of the respondents (N = 1220).

Variables	Answers	N	%
Have you ever heard about generic medicines?	Yes	810	66.4
	No	410	33.6
Do you have knowledge of generic medicines?	Yes	570	46.7
	No	650	53.3
How did you obtain information on generic medicines?	Healthcare practitioner (e.g., pharmacist, physician)	420	34.4
	Media (e.g., internet, television)	150	12.3
	Friends/neighbors	30	2.5
	I have never obtained	410	33.6
	Professional knowledge	210	17.2
Can a generic medicine be produced freely, once the branded product patent protection period has expired, and does it have to be similar to the branded medicine in order to obtain the same therapeutic effect?	Yes	620	50.8
	Don’t know	560	45.9
	No	40	3.3
Do the generic medicines contain the same substance as the branded medicines?	Yes	790	64.8
	Don’t know	350	28.7
	No	80	6.6
Do you think that the generic medicine is of a good quality, when compared to the branded medicine?	Don’t know	410	33.6
	Same	640	52.5
	Lower	130	10.7
	Better	40	3.3
Compared to the branded medicine, do you think that the generic medicine is safe?	Less	90	7.4
	Same	640	52.5
	Don’t know	400	32.8
	More	90	7.4
Do you think that the price of the generic medicine is…	Less than the branded medicines	890	73.0
	Don’t know	290	23.8
	More than the branded medicines	40	3.3

**Table 2:** Practice-related questions and frequency of responses (N = 1220).

Variables	Answers	N	%
Have you already taken generic medicines?	No or don’t know	470	38.5
	Yes	750	61.5
How often do you buy generic medicines?	Never or don’t know	470	38.5
	Always	30	2.5
	Sometimes	360	29.5
	Rarely	260	21.3
	Frequently	100	8.2
Has your physician already prescribed or is prescribing a generic medicine for you?	Never	280	23.0
	Don’t know	400	32.8
	Frequently	40	3.4
	Rarely	190	15.6
	Sometimes	310	25.4
Has your pharmacist proposed you switch to generic medicines?	Sometimes	520	42.6
	Rarely	170	13.9
	Don’t know	190	15.6
	Frequently	150	12.3
	Never	170	13.9
	Always	20	1.6
Have you suggested to your doctor or pharmacist to switch from a branded medicine to a generic medicine?	Sometimes	350	28.7
	Always	50	4.1
	Never	600	49.2
	Rarely	140	11.5
	Frequently	80	6.6
Substitute a branded medicine with a generic because	The generics are cheaper	660	54.1
	The pharmacist recommended it	90	7.4
	I have never used a generic medicine or do not know about it	470	38.5
Did you feel that switching from a brand name to generic medicine changed the outcome of therapy?	I have never used a generic medicine	470	38.5
	No	730	59.8
	Yes	20	1.6
Did you feel side effects when you took generic medicines?	I have never used a generic medicine	470	38.5
	No	690	56.6
	Yes	60	4.9

**Table 3:** Characteristics of the examined group (N = 1220).

Variables	Answers	N	%
Sex	Female	900	73.8
	Male	320	26.2
Marital status	Single	450	36.9
	In an informal relationship	190	15.6
	Married	510	41.8
	Divorced	40	3.3
	Widowed	30	2.5
Place of living	Rural area	150	12.3
	City < 50 thous. residents	70	5.7
	City 50–100 thous. residents	10	0.8
	City 100–500 thous. residents	50	4.1
	City > 500 thous. Residents (Warsaw, Krakow, Wroclaw, Lodz, and Poznan)	940	77.1
Level of education	University	900	73.8
	Secondary	260	21.3
	Vocational	40	3.3
	Primary	20	1.6
Kind of education	Medical	210	17.2
	Non-medical	1010	82.8
Income (monthly after tax) in PLN	<500	30	2.5
	501–1000	60	4.9
	1001–2000	180	14.8
	2001–3000	340	27.9
	3001–4000	250	20.5
	4001–5000	150	12.3
	>5001	210	17.2
Chronic illness other than epilepsy	Yes	430	35.3
	No	790	64.8
Long term use of medication other than antiepileptic drugs	Yes	430	35.3
	No	790	64.8
Healthcare facility where you receive outpatient care	I do not use healthcare	30	2.5
	Public healthcare	390	320
	Public and private healthcare	690	56.6
	Private healthcare	110	9.0

**Table 4:** Opinions and frequency of use of generic medications vs. sociodemographic characteristics (only significant correlations marked X).

Variables	Education (Medical/Non-Medical)	Education Level	Place of Residence	Income
Have you ever heard about generic medicines?	X			X
Do you have knowledge of generic medicines?	X			
How did you obtain information on generic medicines?	X			
Can a generic medicine be produced freely, once the branded product patent protection period has expired, and does it have to be similar to the branded medicine in order to obtain the same therapeutic effect?	X			
Does the generic medicine contain the same substance as the branded medicine?	X			
Do you think that the generic medicine is of a good quality when compared to the branded medicine?	X	X	X	
Compared to the branded medicine, do you think that the generic medicine is safe?	X	X		
Do you think that the price of the generic medicine is…	X	X	X	
Have you already taken generic medicines?		X	X	
How often do you buy generic medicines?		X		

**Table 5:** Correlation between kind of education and knowledge of generic drugs.

**Kind of Education**	**Yes**	**No**	**Sum**	(*p* = 0.000)
Medical	200	10	210	
	95.2%	4.8%		
Non-medical	370	640	1010	
	36.6%	63.6%		
Total	570	650	1220	

**Table 6:** Correlation between kind of education and the answer to the question “Have you ever heard about generic medicines?”.

**Kind of Education**	**Yes**	**No**	**Sum**	(*p* = 0.002)
Medical	200	10	210	
	95.2%	4.8%		
Non-medical	610	400	1010	
	60.4%	39.6%		
Total	810	410	1220	

**Table 7:** Correlation between kind of education and the answer to the question “Can a generic medicine be produced freely once the branded product’s patent protection period has expired, and does it have to be similar to the branded medicine in order to obtain the same therapeutic effect?”.

**Kind of Education**	**Yes**	**Don’t Know**	**No**	**Sum**	*p* = 0.006
Medical	170	30	10	210	
	81%	14.3%	4.7%		
Non-medical	450	530	30	1010	
	44.5%	52.5%	3%		
Total	620	560	40	1220	

**Table 8:** Correlation between kind of education and the answer to the question “Does the generic medicine contain the same substance as the branded medicine?”.

**Kind of** **Education**	**Yes**	**Don’t Know**	**No**	**Sum**	*p* = 0.000
Medical	200	10	0	210	
	95.2%	4.8%	0.00%		
Non-medical	590	340	80	1010	
	58.4%	33.7%	7.9%		
Total	790	350	80	1220	

**Table 9:** Correlation between income (monthly after tax) in PLN and knowledge of generic drugs.

**Income (Monthly after Tax) in PLN**	**Yes**	**No**	**Sum**	*p* = 0.05
2001–3000	250	80	330	
	75.8%	24.2%		
4000–5000	110	40	150	
	73.3%	26.7%		
3001–4000	190	60	250	
	76.0%	24.0%		
1001–2000	50	120	170	
	29.4%	70.6%		
>5001	140	70	210	
	66.7%	33.3%		
501–1000	40	20	60	
	66.7%	33.3%		
<500	20	10	30	
	66.7%	33.3%		
Total	810	410	1220	

## Data Availability

All data are available from the corresponding author.
